# *Onchocerca jakutensis* ocular infection in Poland: a new vector-borne human health risk?

**DOI:** 10.1186/s13071-020-3925-6

**Published:** 2020-02-12

**Authors:** Maria Wesołowska, Hanna Zając-Pytrus, Aleksander Masny, Wiktoria Pytrus, Brygida Knysz, Elzbieta Golab, Rusłan Sałamatin

**Affiliations:** 1 Department of Biology and Medical Parasitology, Wrocław Medical University, Wrocław, Poland; 2 Department and Clinic of Ophthalmology, Wrocław Medical University, Wrocław, Poland; 3 Ophthalmology Clinical Centre SPEKTRUM, Wrocław, Poland; 4 Department of Virology, National Institute of Public Health – National Institute of Hygiene, Warsaw, Poland; 5 Department of Infectious Diseases, Liver Diseases and Acquired Immune Deficiencies, Wrocław Medical University, Wrocław, Poland; 6 Department of Parasitology and Vector-Borne Diseases, National Institute of Public Health – National Institute of Hygiene, Warsaw, Poland; 7 Department of General Biology and Parasitology, Medical University of Warsaw, Warsaw, Poland

**Keywords:** *Onchocerca jakutensis*, Human, Filarioidea, Onchocercidae, Vector-borne helminths, Poland

## Abstract

**Background:**

Zoonotic onchocerciasis is a vector-borne disease, which involves many animal species, including large ungulates, boars, dogs, and sporadically, humans. So far, 39 cases of zoonotic onchocerciasis have been reported worldwide, 30 of which have been found in the last 20 years. *Onchocerca* nematodes are transmitted to humans by blood-sucking vectors during a blood meal. The following species have been responsible for zoonotic infections: *Onchocerca cervicalis*, *O. dewittei japonica*, *O. gutturosa*, *O. jakutensis* and *O. lupi*. In humans, the worms have usually been found in the subcutaneous tissues where they form subcutaneous nodules, induce inflammation of musculature, or penetrate the eye. Thirteen ocular zoonotic onchocerciasis cases have been reported so far. In the eye, nematodes were localized in the subconjunctival space, anterior chamber and within the vitreous body.

**Methods:**

In a 39-year-old male patient, a writhing worm in the vitreous body of the left eye was detected and surgically removed. Laboratory identification of the worm was based on macroscopic and molecular identification, based on sequencing of the cytochrome *c* oxidase subunit 1 gene (*cox*1). Phylogenetic analysis of the first 250 nucleotide sequences showing the highest levels of similarity with the present isolate in a BLAST analysis was performed.

**Results:**

Here, we report the first case worldwide of human ocular infection with *O. jakutensis*, a natural parasite of red deer. By exploiting a PCR assay, we detected the sequence almost identical to *O. jakutensis* (GenBank: KT001213.1; positions 1–650) with a single mismatch G/A at position 622. The sequence reported in this paper was deposited in the GenBank database under the accession number MK491767.

**Conclusions:**

Our case together with the previous case reports indicate that zoonotic *Onchocerca* worms exhibit no tissue specificity and an eye infection has been described in over one third of human zoonotic onchocerciasis cases. In terms of the growing number of cases of zoonotic onchocerciasis in Europe, the USA and Japan, attention should be paid to the diagnosis of subcutaneous nodules and eye infestations.
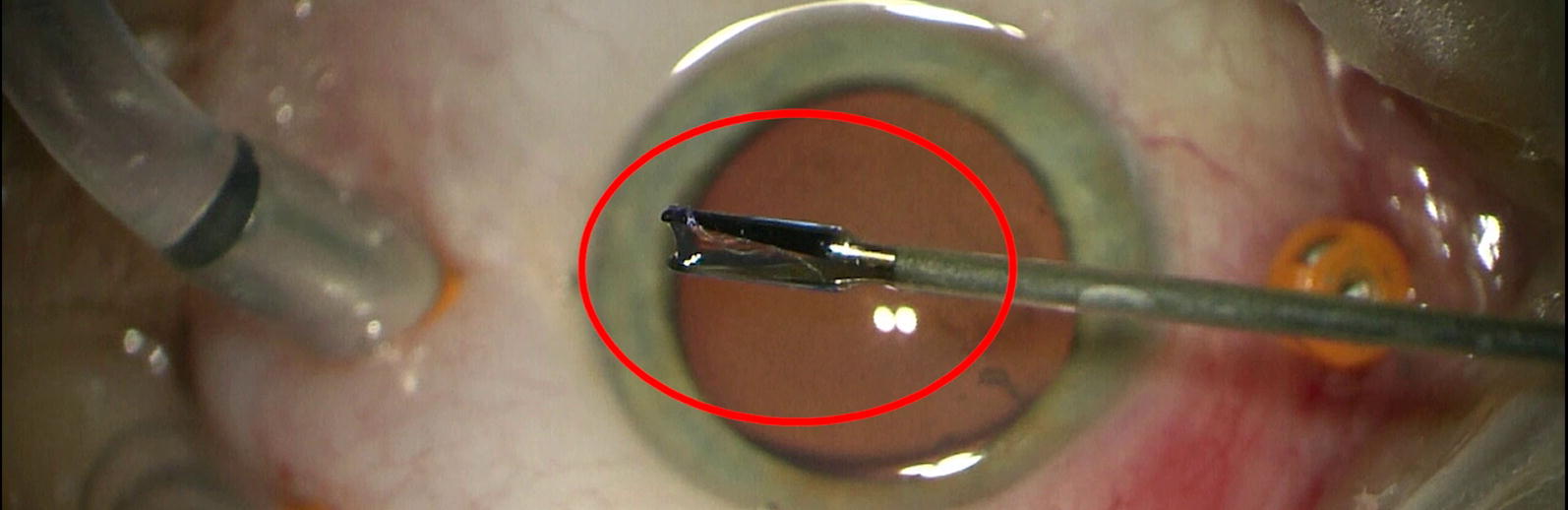

## Background

Zoonotic onchocerciasis is a vector-transmitted disease caused by nematodes of the genus *Onchocerca* (Spirurida: Onchocercidae) which are commonly transmitted between wild and domestic animals and occasionally infect humans [1]. Most cases of human zoonotic onchocerciasis (other than caused by *O. volvulus*, which is an exclusively human parasite) were reported in Europe, the USA and Japan. In recent years an increased incidence of the disease has been observed around the world [2]. So far 39 cases have been described worldwide; 22 females and 17 males, aged from 2 to 78 years were infected. Most of these cases were reported after 2000, indicating either an increasing number of cases or better diagnostics or both. At present, the following five zoonotic *Onchocerca* spp. have been identified in humans: *O. lupi*, *O. gutturosa*, *O. cervicalis*, *O. dewittei japonica* and *O. jakutensis. Onchocerca* spp. are transmitted to humans by blood-sucking vectors, such as horse flies, blackflies and biting midges during a blood meal [3]. In humans, the worms have been found in the subcutaneous tissue forming subcutaneous nodules, causing inflammation of musculature, or penetrated the eye.

Herein we report the first case worldwide of human ocular infection with *O. jakutensis* found in the vitreous cavity.

## Methods

### Case presentation

A 39-year-old male patient was admitted in the Centre of Clinical Ophthalmology Spectrum in Wrocław, Poland, in February 2018 due to impaired vision and a moving floater in his left eye he had experienced for a month. No information about the time of the infection was available. General physical examination results were normal and there was no history of any other diseases. In the blood test, no eosinophilia or microfilaria were found. Other routine blood tests did not reveal any abnormalities. No immune deficiency was identified.

**Fig. 1:**
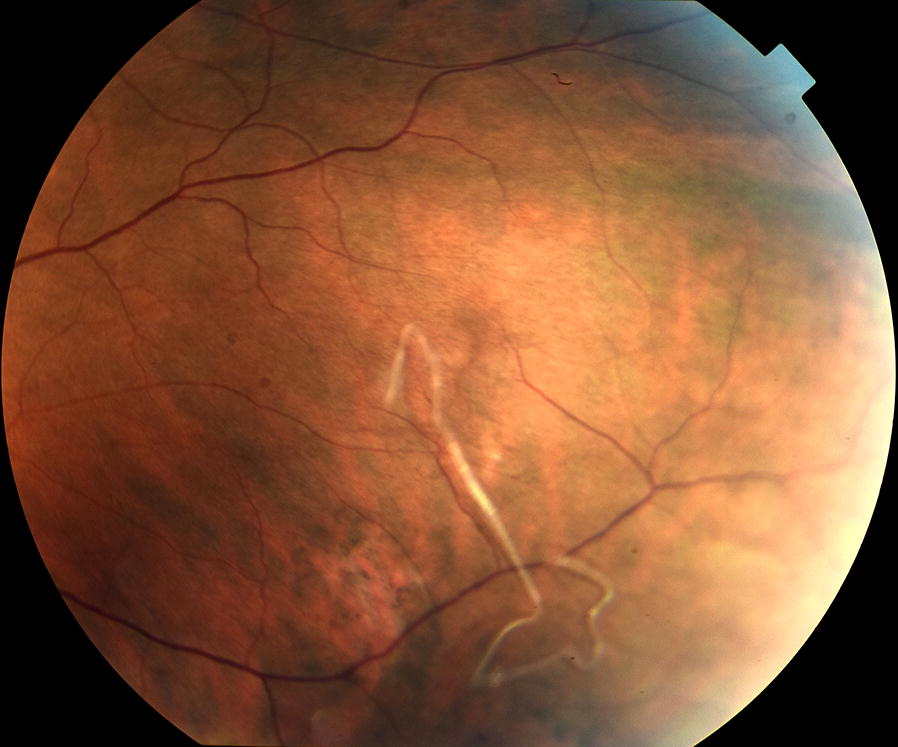
Immature nematode of *Onchocerca jakutensis* floating in the vitreous body of the left eye. The worm measured about 25 mm and was removed *via* pars plana vitrectomy by the two hands technique

The ophthalmic examination revealed a writhing worm in the vitreous body of the left eye (Fig. 1, Additional file 1: Video S1). The patient did not feel pain, but the actively motile worm impaired correct vision. The anterior and posterior segments showed no signs of inflammation. Visual acuity (VA) was 20/20, intraocular pressure (IOP) was normal. The right eye was completely normal. The patient underwent 23-gauge vitrectomy. A fourth sclerotomy was needed for a light-pipe and bimanual technique necessary to capture and remove the live nematode tightly attached to the surrounding tissues for further examination. During the surgical removal the parasite moved very fast, which made it difficult to catch and it was accidentally cut. The postoperative follow-up was uneventful. VA remained 20/20. At present the patient suffers from a tiny floater in the vitreous body to incomplete vitrectomy. Complete vitrectomy was not carried out because of the risk of cataract in the young patient with an accommodating lens. The patient lives in a rural area surrounded by forests in Western Poland (51° 37′ 03″ N, 15° 18′ 53″ E; 98 m height above sea level) near the Polish-German border. He has never travelled abroad so the case is autochthonous, and it is likely that he became infected with a locally transmitted filarial species. He did not report an insect bite in the ocular, periocular or facial region.

### Identification of the worm

Laboratory identification of the worm was based on macroscopic and molecular identification. The DNA was isolated and purified using the Genomic Mini Kit, A&A Biotechnology. Two PCR reactions were performed: PCR1 with primer pair RepIm-F0 (5′-TCA GAT TAG TAT GTT TGT TTG AAC TTC TTA TTT-3′) and RepIm-R0 (5′-ACA GCA ATC CAA ATA GAA GCA AAA GT-3′) [4]; and PCR2 with primer pair H14FilaCOIFw (5′-GCC TAT TTT GAT TGG TGG TTT TGG-3′) and H14FilaCOIRv (5′-AGC AAT AAT CAT AGT AGC AGC ACT AA-3′) [5]. Amplified products of both PCR reactions were then subjected to automated Sanger sequencing.

The obtained nucleotide sequences were assembled using CLC MainWorkbench ver. 6.9.1 software and analysed using NCBI BLAST [6]. The first 250 nucleotide sequences showing the highest level of similarity in the NCBI BLAST analysis were used for phylogenetic tree building. Bayesian inference was performed using MrBayes version 3.2.7a on: the 250 sequences retrieved from GenBank (Additional file 2: Table S1), the sequence obtained in our study and a sequence for *Filaria latala* (GenBank: KP760186.1) used as the outgroup [7].

## Results

The removed worm was heavily twisted and had one end broken, so the length of the remaining part was about 25 mm; the diameter was about 120 μm. Its internal organs were collapsed, and the cuticle was distorted by surgical tools, which made it difficult to assess the morphological features. Therefore, after the preliminary measurements we decided to perform molecular diagnostics.

In the PCR1, a 309 bp DNA sequence was obtained (250 bp excluding primers), which was identical to a fragment of the sequence KT001213.1 (positions 331–580) of a specimen of *O. jakutensis* obtained from *Cervus elaphus* in Austria [8].

The product of the PCR 2 was a 674-bp DNA sequence (650 bp excluding forward primer sequence). The obtained sequence was almost identical to that for *O. jakutensis* KT001213.1 (positions 1–650) with a single mismatch G/A at position 622.

**Fig. 2:**
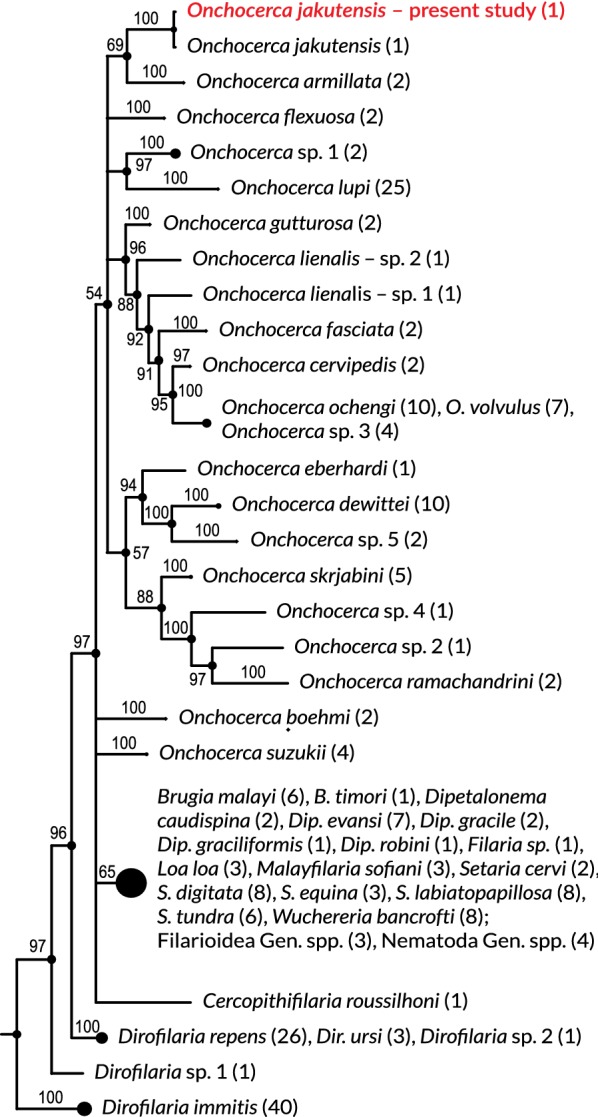
Bayesian inference tree based on the 517-bp fragment of 250 distinct sequences of the *cox*1 gene for 50 filarial species (outgroup not shown). The number of sequences from each species is shown in parentheses. The Bayesian posterior probabilities are shown adjacent to branch nodes

The phylogenetic relationships between the newly generated sequence and the 250 nucleotide sequences of the cytochrome *c* oxidase subunit 1 gene representing 50 filarial species are presented in Fig. 2.

## Discussion

Human zoonotic ocular onchocerciasis is very rare. A review of the literature revealed 13 cases of eye infection with zoonotic *Onchocerca* spp., which accounts for 33% of all onchocerciasis cases worldwide. Most cases of zoonotic ocular *Onchocerca* infection were caused by *O. lupi*, less frequently by *O. gutturosa* and *O. cervicalis* [2, 5, 6]. To the best of our knowledge, we report the first case of *O. jakutensis* ocular infection*. Onchocerca jakutensis* is a common parasite of red deer detected in the Russian Federation [9], Switzerland [10], Italy [1], Germany [11], the Czech Republic [12], the Slovak Republic [12] and Poland [9]. A study by Demiaszkiewicz [13] has shown that *O. jakutensis* parasitize red deer in several regions of Poland, among others, in the south-west part, where patient described in the present report lives. In the case of the patient described here, alive, fast moving worm was located in the vitreous body. According to the literature, the *Onchocerca* nematodes belonging to the other species, alive or dead, have also been found in the anterior chamber of the eyeball, in the subconjunctival space, and caused conjunctival nodule formation [14–16].

The literature describes only one case of human infection with *O. jakutensis*. In 2007, this parasite was detected in Austria in a 57-year-old woman with nodules on the neck and face [17]. In red deer, *O. jakutensis* is usually found in the tissues of the outer thigh and the caudal part of the back thus, the location of both human cases, i.e. an eye infection and neck and face nodules are unusual for this species.

There is little opportunity to treat onchocerciasis, ivermectin being the recommended treatment. The efficacy of ivermectin in relation to larvae has been confirmed but it affects adult worms weakly. Therefore, the most effective method of treatment is surgical removal of the palpable nodules containing the worms. The difficulty in removing an intact worm from the eye reduces the chances of obtaining a good quality sample for morphological identification of the worm. Therefore, molecular identification is necessary to establish the correct diagnosis. In ocular onchocerciasis, surgical removal of the worm decreases eye complications [18].

## Conclusions

Onchocerciasis may be expected in generally healthy people. It is not clear whether the number of zoonotic onchocerciasis cases is growing, or the availability of novel diagnostic tools is increasing the number of the confirmed and correctly identified onchocerciasis cases. Humans cannot completely eliminate their exposure to vector-borne zoonotic diseases. If zoonotic *Onchocerca* affects animals inhabiting the same areas as people do, they may become infected too. To our knowledge, the presented case is the first finding of *O. jakutensis* in the human eye. Both physicians and laboratory staff should be aware of the existence of zoonotic onchocerciasis in their countries.

## Supplementary information

**Additional file 1: Video S1.** *Onchocerca jakutensis* removal *via* pars plana vitrectomy by two hands technique.

**Additional file 2: Table S1.** The sequences used for phylogenetic analysis.

## Data Availability

The data supporting the conclusions of this article and all manuscripts included in the literature review are available within the article and its additional files. The sequence generated in this study was deposited in the GenBank database under the accession number MK491767.

## Abbreviations

IOP: Intraocular pressure
PCR: Polymerase chain reaction
VA: Visual acuity
