# Supplementary material for: Onchocerca jakutensis ocular infection in Poland: a new vector-borne human health risk?
Source: Parasit Vectors. 2020 Feb 12;13:61. doi: 10.1186/s13071-020-3925-6 (PMC7017525; doi:10.1186/s13071-020-3925-6)
Supplement: Supplementary file 2 — Additional file 2: Table S1. The sequences used for phylogenetic analysis. [file 13071_2020_3925_MOESM2_ESM.docx]

**Additional file 2: Table** **S1.** **The** **sequences** **used** **for** **phylogenetic** **analysis.**

|  | **Species** | **GenBank** **accession** **number** |
| --- | --- | --- |
| 1 | *Brugia* *malayi* | AF538716.1, MK250713.1, MK250714.1, MK250720.1, MK250728.1, MK250733.1 |
| 2 | *Brugia* *timori* | AP017686.1 |
| 3 | *Cercopithifilaria* *roussilhoni* | AM749264.1 |
| 4 | *Dipetalonema* *caudispina* | KP760177.1, KP760178.1 |
| 5 | *Dipetalonema* *evansi* | KR184801.1, KR184805.1, KR184807.1, KR184808.1, KR184809.1, KR184813.1, KR184816.1 |
| 6 | *Dipetalonema* *gracile* | KP760179.1, KP760180.1 |
| 7 | *Dipetalonema* *graciliformis* | KP760182.1 |
| 8 | *Dipetalonema* *robini* | KP760183.1 |
| 9 | *Dirofilaria* *immitis* | AJ271613.1, AJ537512.1, DQ358815.1, EU159111.1, KF692101.1, KM452920.1, KR870344.1, LC107816.1, MH920260.1, MK250715.1, MK250717.1, MK250718.1, MK250719.1, MK250721.1, MK250723.1, MK250724.1, MK250727.1, MK250731.1, MK250734.1, MK250735.1, MK250736.1, MK250736.1, MK250737.1, MK250738.1, MK250740.1, MK250741.1, MK250742.1, MK250744.1, MK250745.1, MK250746.1, MK250747.1, MK250748.1, MK250749.1, MK250750.1, MK250751.1, MK250756.1, MK250757.1, MK250758.1, MK250759.1, MK250760.1 |
| 10 | *Dirofilaria* *repens* | AB973225.1, AJ271614.1, AM749230.1, AM749231.1, AM749232.1, AM749233.1, AM749234.1, DQ358814.1, JF461458.1, KF692102.1, KP760185.1, KR071802.1, KR780980.1, KR998257.1, KR998259.1, KT901783.1, KX265047.1, KX265048.1, KX265049.1, KY085963.1, KY828978.1, KY828979.1, MF695085.1, MH541831.1, MH780816.1, MH780817.1, MK210632.1 |
| 11 | *Dirofilaria* sp. 1: „GM-2017” | KY085963.1 |
| 12 | *Dirofilaria* sp. 2: „hongkongensis” | KX265050.1 |
| 13 | *Dirofilaria* *ursi* | KY828980.1, KY828981.1, KY828982.1 |
| 14 | *Filaria* *latala* | KP760186.1 |
| 15 | *Filaria* sp.: „FZ-2014” | KJ612514.1 |
| 16 | Filarioidea Gen. sp. 1: „JA-2012” | JX870433.1 |
| 17 | Filarioidea Gen. sp. 2: „DBB-2016-III” | LC107819.1 |
| 18 | Filarioidea Gen. sp. 3: „Dan_Ex-75” | LC107949.1 |
| 19 | *Loa* *loa* | AJ544875.1, HQ186250.1, KP760194.1 |
| 20 | *Malayfilaria* *sofiani* | KX944563.1, KX944564.1, KX944565.1 |
| 21 | Nematoda Gen. sp. 1: „MC-MOTU-1” | AM749287.1, AM749288.1 |
| 22 | Nematoda Gen. sp. 2: „MC-MOTU-2” | AM749289.1, AM749290.1 |
| 23 | *Onchocerca* *armillata* | KP760200.1, KX853322.1 |
| 24 | *Onchocerca* *boehmi* | KX853323.1, KX898458.1 |
| 25 | *Onchocerca* *cervipedis* | AJ271616.1, KX853324.1 |
| 26 | *Onchocerca* *dewittei* | AB518689.1, AB518690.1, AB518691.1, AB518692.1, AB518872.1, AB518873.1, AB518874.1, AB518875.1, AM749266.1, KP760203.1 |
| 27 | *Onchocerca* *eberhardi* | AM749268.1 |
| 28 | *Onchocerca* *fasciata* | JQ316672.1, MG188678.1 |
| 29 | *Onchocerca* *gutturosa* | AJ271617.1, KP760201.1 |
| 30 | *Onchocerca* *jakutensis* | KT001213.1 |
| 31 | *Onchocerca* *lienalis* – sp.1 | KX853325.1 |
| 32 | *Onchocerca* *lienalis* – sp. 2 | KX853326.1 |
| 33 | *Onchocerca* *lupi* | AJ415417.1, EF521409.1, EF521410.1, HQ207644.1, JF758473.1, JF758474.1, JX080028.1, JX080029.1, JX080030.1, JX080031.1, JX183106.1, KC686701.1, KC686702.1, KC763786.1, KP283476.1, KP283477.1, KX132091.1, KX853327.1, KX853328.1, KX853329.1, KX853330.1, KX853331.1, KX853332.1, MF464462.1, MG677940.1 |
| 34 | *Onchocerca* *ochengi* | AP017694.1, NC_031891.2, KX181289.2, AP017693.1, KC167350.1, AJ271618.1, KC167358.1, KP760202.1, KC167351.1, KX181290.2 |
| 35 | *Onchocerca* *ramachandrini* | KC167356.1, KC167357.1 |
| 36 | *Onchocerca* *skrjabini* | AM749269.1, AM749270.1, AM749271.1, AM749272.1, AM749274.1 |
| 37 | *Onchocerca* sp. 1: „ALDA-2017” | MG029460.1, MG029461.1 |
| 38 | *Onchocerca* sp. 2: „type A” | AB518876.1 |
| 39 | *Onchocerca* sp. 3: („Siisa”, „AY-2014”) | KC167352.1, KC167353.1, KC167354.1, KF999650.1 |
| 40 | *Onchocerca* sp. 4: „JJP-2019” | MK541848.1 |
| 41 | *Onchocerca* sp. 5: „wild boar” | AB518693.1, AB518694.1 |
| 42 | *Onchocerca* *suzukii* | AM749275.1, AM749276.1, AM749277.1, KX853333.1 |
| 43 | *Onchocerca* *volvulus* | AF015193.1, AM749284.1, AM749285.1, AP017695.1, KC167355.1, KT599912.1, MH190075.1 |
| 44 | *Onchocerca* *flexuosa* | AP017692.1, HQ214004.1 |
| 45 | *Setaria* *cervi* | JF800924.1, MK360913.1 |
| 46 | *Setaria* *digitata* | AM886173.1, EF174423.1, EF174425.1, EF174426.1, EF174427.1, GU138699.1, KY284626.1, MN078131.1 |
| 47 | *Setaria* *equina* | AJ544873.1, MK541847.1, MK629666.1 |
| 48 | *Setaria* *labiatopapillosa* | AJ544872.1, KP760208.1, KX570601.1, MF589581.1, MF589582.1, MF589583.1, MF589584.1, MF589585.1, NC_044071.1 |
| 49 | *Setaria* *tundra* | AJ544874.1, AM749298.1, DQ097309.1, KF692103.1, KF692104.1, KF692105.1, KM452922.1, KP760209.1, KU508983.1, KU508984.1, KU508985.1, KX599455.1, KX599456.1, MF695086.1, MF695088.1, MF695089.1, MF695090.1, MF695091.1, MF695092.1, MF695094.1, MF695095.1, MF695096.1, MH541830.1, MK360914.1, MK360915.1 |
| 50 | *Wuchereria* *bancrofti* | AJ271612.1, AM749235.1, AP017705.1, HQ184469.1, JF775522.1, JN367461.1, JQ316200.1, KY883763.1 |
